# Antimicrobial Effects of *Sophora flavescens* Alkaloid*s* on Metronidazole-Resistant *Gardnerella vaginalis* in Planktonic and Biofilm Conditions

**DOI:** 10.1007/s00284-023-03378-x

**Published:** 2023-06-29

**Authors:** Linyuan Fan, Zhaohui Liu, Zhan Zhang, Huihui Bai

**Affiliations:** 1 Department of Gynecology, Beijing Obstetrics and Gynecology Hospital, Capital Medical University, Beijing Maternal and Child Health Care Hospital, No. 251, Yaojiayuan Road, Chaoyang District, Beijing, 100026 China; 2 Department of Clinical Laboratory, Beijing Obstetrics and Gynecology Hospital, Capital Medical University, Beijing Maternal and Child Health Care Hospital, Beijing, 100026 China

## Abstract

Bacterial vaginosis (BV) is a common infectious disease of the lower female reproductive tract, which is characterized by the augmentation of anaerobic bacteria. *Gardnerella (G.) vaginalis* plays a predominant role in BV recurrence relating to its higher virulence potential and biofilm formation ability. With the increased proportion of metronidazole-resistant *G. vaginalis,* controlling resistance to metronidazole and finding more effective drugs became a major concern. In this study, 30 clinical strains were cultured from the vaginal secretions of BV patients, followed by PCR and 16S rDNA sequencing identification. According to the CLSI guidelines for anaerobic drug sensitivity testing, 19 strains were identified as metronidazole-resistant (minimum inhibitory concentration, MIC ≥ 32 μg/mL), of which 4 clinical strains were observed to be strong biofilm producer and the final minimum biofilm inhibitory concentration (MBIC) of metronidazole was increased to 512 μg/mL. *Sophora flavescens* Alkaloids (SFAs), a traditional chinese medicine, could not only inhibit the growth of metronidazole-resistant *G. vaginalis* in planktonic (MIC: 0.3125–1.25 mg/mL), but also eliminate the biofilm formation (MBIC: 0.625–1.25 mg/mL). In the high-magnification scanning electron, it was observed that the morphology of biofilm changed from a thick to flaky shape and was nearly depleted. These results indicate that SFAs could not only inhibit the growth of metronidazole-resistant *G. vaginalis*in planktonic and biofilm levels, but also destroyed the biofilm morphology and microstructure, which may contribute to the prevention of BV recurrence.

**Supplementary Information:**

The online version contains supplementary material available at 10.1007/s00284-023-03378-x.

## Introduction

Bacterial vaginosis (BV) is an infectious disease of the lower female reproductive tract and frequently affects women of childbearing age. It causes an abnormal proliferation of anaerobic microorganisms, primarily *Gardnerella (G.) vaginalis* and further leads to a decrease or disappearance of *Lactobacillus vaginalis* altering the composition of the vaginal flora [1, 2]. The reproductive health of millions of women in the world is seriously affected by BV every year. According to published data, the morbidity rate for BV is 5–15% in white women and 45–55% in black women [3].BV causes adverse pregnancy outcomes, such as spontaneous abortion, premature delivery, amniotic fluid infection, puerperal endometritis, cesarean incision infection, and perinatal complications [4].The recurrence rate of BV is high and gradually increases with time. Disease relapse has been observed in one-third of patients at three months after treatment. Moreover, persistent BV infection and recurrence can also increase the risk of trichomonas vaginitis, vulvovaginal candidiasis, cervical cancer, and human immune deficiency virus infection [5].

*G. vaginalis* is a facultative anaerobic Gram-negative hemophilic bacillus that is difficult to culture and can be transmitted by sexual contact. *G. vaginalis* adheres tightly to the surface of vaginal epithelial cells and can form densely clustered biofilm with potent cytotoxicity against vaginal epithelial cells. Other BV-related bacteria are less able to adhere to vaginal epithelial cells and form biofilm. Therefore, *G. vaginalis* plays a predominant role in BV occurrence [6]. Also, *G. vaginalis* can form a specialized adhesive biofilm that competes with lactobacillus and coexists with normal dormant vaginal anaerobic bacteria to increase their numbers, which also contribute to BV. Current studies have demonstrated that biofilm formation plays a critical role in the pathogenesis of BV. The occurrence and failure of BV treatment is related to *G. vaginalis* biofilm formation in the vagina [7].

Currently, BV is primarily treated with systemic or local administration of anti-anaerobic antibiotics. As the first-line of therapy for BV, metronidazole was recommended by Centers for Disease Control and Prevention (CDC). While the recurrence rate of BV is as high as 33% and 49–66% within three months and one year after treatment, respectively [8]. Metronidazole-resistant *G. vaginalis* might be one reason for BV recurrence and treatment failure. *Sophora flavescens* alkaloids (SFAs), a traditional chinese medicine, is extracted from the *Sophora flavescent*. Currently, more than 20 alkaloids have been isolated and identified from *Sophora flavescens* alkaloids, and the main active of these components is matrine and oxymatrine. SFAs have multiple antibacterial and anti-inflammatory activities, as well as pharmacological antipruritic potential [9]. Previous studies showed that SFAs (such as *Sophora flavescens* gel) has the ability to restore vaginal microbiota and mucosal repair, resulting in decreased recurrence of vaginitis effects [10]. Furthermore, researchers found that SFAs can inhibit bacterial biofilm formation of *Staphylococcus epidermidis* (*S. epidermidis*) by exhibiting the auto-inducer molecule (AI-2) activity [11]. However, the antibacterial activity and mechanism of SFAs against *G. vaginalis*, especially the metronidazole-resistant clinical strains, has not been reported. This study evaluated the susceptibility of planktonic *G. vaginalis* and the biofilm formation of metronidazole-resistant strains to SFAs, which will provide an experimental data of SFAs in BV.

## Materials and Methods

### High Performance Liquid Chromatography Analysis

Matrine and oxymatrine were the main contents of SFAs and were analyzed by HPLC methods. The standard control sample of matrine and oxymatrine were both purchased from National Institute for the Control of Pharmaceutical and Biological Products (Beijing, China), the chromatographic analyses were implemented on the Agilent UPLC system (Shimadzu, Japan), and the Agilent XDB C18 5 μm (4.6 × 150 mm) was used for the chromatographic separation. Mobile phase consisted of solvent A (10% water) and B (90% methanolsolution), and the flow rate was set to 1 mL/min with the injection volume was 4 μL. A sample of 1 g SFAs was put into a 100 mL volumetric flask with constant volume of methanol to obtain 10 mg/mL test solution, the control sample matrine and oxymatrine were used as the referenced sample. The operating parameters were as follows: both the reference and test samples were prepared into different gradient solutions with the same concentration. The reference substance and the test substance solution were injected, respectively at the same time to obtain the peak area of the different component. Since the response value factor (*F*) of each component is unchanged, therefore, the concentration of test sample can be calculated according to *F* value. *F* is calculated as follows: *F* = reference concentration/reference peak area = test concentration / test solution peak area. So the oxymatrine content = [(concentration × dilution ratio) / test product weight sample volume] × 100%. Results are presented as mean ± SD of the mean of at least triplicates.

### Isolation and Culture of *G. vaginalis* Strains

The strains used in this study were isolated from vaginal secretions of bacterial vaginosis patients in the Gynecology Clinic as per the following procedure. The vaginal secretions were obtained from one-third of the vaginal wall using a sterile cotton swab and applied to a Casman agar plate (Beijing AOBOX Biotechnology Co., Ltd.).Then the agar plate was placed into an aerobic bag and incubated with 5% CO_2_ at 37 °C for 48 h. Round, needle-like, and translucent single colonies were selected and applied in line to a new Casman agar plate followed by anaerobic culture for 24–48 h. After three generations of pure cultures, single colonies were collected, mixed evenly with 30% glycerol, and stored at − 80 °C.

### Identification of *G. vaginalis* Strains

The clinically isolated strains were identified using colony polymerase chain reaction (PCR) and 16S rDNA sequencing. 16S rDNA gene hypervariable V1–V3 region was amplified using the primers 27 F (5ʹ-AGAGTTTGATCC TGGCTCAG-3ʹ) and 1492 R (5ʹ-GGTTACCTTGTTAGACTT-3ʹ).The protocol used was as follows: total volume of the PCR mixture was 50 μL, and the reaction conditions included pre-denaturation at 95 °C for 5 min, denaturation at 95 °C for 30 s, then annealing and extension at 72 °C for 80 s and the cycle was repeated 33 times. Then part of the sample is taken for gel electrophoresis test to verify the safe of this PCR operation. All of the PCR amplification products were sent to Beijing SinoGenoMax Co., Ltd. for sequencing, and the sequences of the splicing results were compared with the bacterial 16S rDNA gene sequences in GenBank and National Center for Biotechnology Information (NCBI) data library for identification and confirmation of *G. vaginalis*. *Bacteroides fragilis* ATCC 25285 was used as a control for the test carried out under anaerobic conditions.

### Antimicrobial Susceptibility Testing

*Sophora flavescens* Alkaloids (NMPN Z20050058) was provided by Guiyang Xintian PharmaceuticalCo., Ltd. (Guizhou, China) and metronidazole (purity: 99.97%) was purchased from National Institute for the Control of Pharmaceutical and Biological Products (Beijing, China). SFAs was dissolved in ethanol with concentration of 400 mg/mL and metronidazole dissolved by sterile distilled water with 2.56 mg/mL concentration. The antimicrobial susceptibility activity was detected by the microdilution broth method as described by Sutyak Nollwith minor modifications. Briefly, the antimicrobials were diluted (a series of twofold dilutions) with an appropriate volume of fresh brain heart infusion medium (Beijing AOBOX Biotechnology Co., Ltd.) in 96-well culture plate. The final concentration of the SFA was 0.039–20 mg/mL and metronidazole was 0.125–128 μg/mL. An equal proportion of ethanol solution at the highest final concentration of 5% and sterile distilled water were served as the negative control, respectively. Sterile distilled water served as the negative control. *G. vaginalis* and control were cultured until they achieved the logarithmic-phase and were diluted with BHI to the final 5 × 10^6^ CFU/mL. From the diluted bacterial cells, 100 μL was transferred in the wells containing predetermined concentrations of antimicrobial. The inoculated plate was placed into an anaerobic chamber and incubated under 5% CO_2_ at 37 °C for 48 h. The lowest antibiotic concentration yielding marked reduction to no growth was read as the Minimum Inhibitory Concentration (MIC). According to the 2012 and 2018 Clinical and Laboratory Standards Institute (CLSI) guidelines for anaerobic drug sensitivity testing, metronidazole MIC was evaluated as follows: Sensitive: MIC ≤ 8 μg/mL; Intermediate: MIC = 16 μg/mL; Resistant: MIC ≥ 32 μg/mL [12, 13].

As previous described, after the plate was inoculated for 48 h, 100 μL of the bacterial solution was separately taken from the non-turbidity and control wells, then were evenly applied to a drug-free solid medium, followed by anaerobic culture for 48 h. The drug concentration in the well where the total bacterial count declined by 99.9% or more was compared against the control well to reveal the minimum bactericidal concentration (MBC).

### Bacterial Biofilm Formation Assay

To develop the biofilm formation level of *G. vaginalis* clinical strains, a starting inoculum of 5 × 10^6^ CFU/mL of prepared bacterial suspension in the BHI medium, was planted in 96-well culture plate. The microplate was incubated anaerobically for 48 h. Crystal violet staining (Beijing AOBOX Biotechnology Co., Ltd.) was used to quantify the total amount of biofilm biomass. After the incubation period, each well was gently washed twice with 200 μL of phosphate buffered saline to remove the non-adhered bacteria, and dried for 15 min. The dried biofilm was then stained with 200 μL crystal violet (1%, w/v, Sigma) and incubated for 30 min. Finally, the well was rinsed with phosphate buffered saline three times to completely remove unbound crystal violet and the combined was decolorized with 200 μL 95% alcohol for 5 min. Then the liquid was moved to a new 96-well microtitre plate and absorbance at 595 nm was measured by a microplate reader (Bio-Rad Laboratories, Hercules, CA, USA). The ability of biofilm formation was evaluated according to optical density (OD) value. We defined the cut-off OD (ODc) for microtiter-plate test as three standard deviations above the mean OD of the negative control. The biofilm formation capabilities of clinical strains were described as below: OD ≤ 2 × ODc, weakly biofilm producer; 2 × ODc ≤ OD ≤ 4 × ODc, moderately biofilm producer; OD ≥ 4 × ODc, strongly biofilm producer [14, 15]. In this study, 4 strains were found to have strong biofilm-forming ability and were selected for subsequent experiments.

### Determination of the Minimal Biofilm Inhibitory Concentration (MBIC)

The inhibitory concentration of *Sophora flavescens* Alkaloids against *G. vaginalis* biofilm formation was determined as above. The logarithmic-phase bacteria was cultured with antimicrobial-containing medium and anaerobically cultured for 48 h. *G. vaginalis* biofilm was detected by violet solution and the minimum biofilm inhibition concentration (MBIC) was defined as the lowest concentration of an antibiotic that completely inhibited the growth of microorganisms compared with control.

### Observation of Biofilm Microstructure Using Scanning Electron Microscopy (SEM)

The morphology and structure of biofilm changes were described by scanning electron microscopy examination. Sterile cover glass was placed at the bottom of each well of a 24-well plate, and bacteria were inoculated into each well glass, followed by anaerobic culture for 48 h to form biofilm. Then the cover glasses were removed, washed three times with phosphate buffered saline, fixed with 2% cold glutaraldehyde for 15 h, vacuum-dried for 72 h, and plated with gold. The morphological feature of biofilm was observed using a scanning electron microscope (HITACHI S-3400, Japan).

### Statistical Analysis

GraphPad Prism 6.0 software (GraphPad Software, Inc., La Jolla, CA) was used for statistical analysis. Non-parametric Mann-Whimey *U* test was used for comparison because the data did not conform to normal distribution. *P* < 0.05 (two-tailed) indicated a statistically significant difference .

## Results

### Quantitative analysis of the Oxymatrine and Matrine in *Sophora flavescens* Alkaloids

**Fig. 1:**
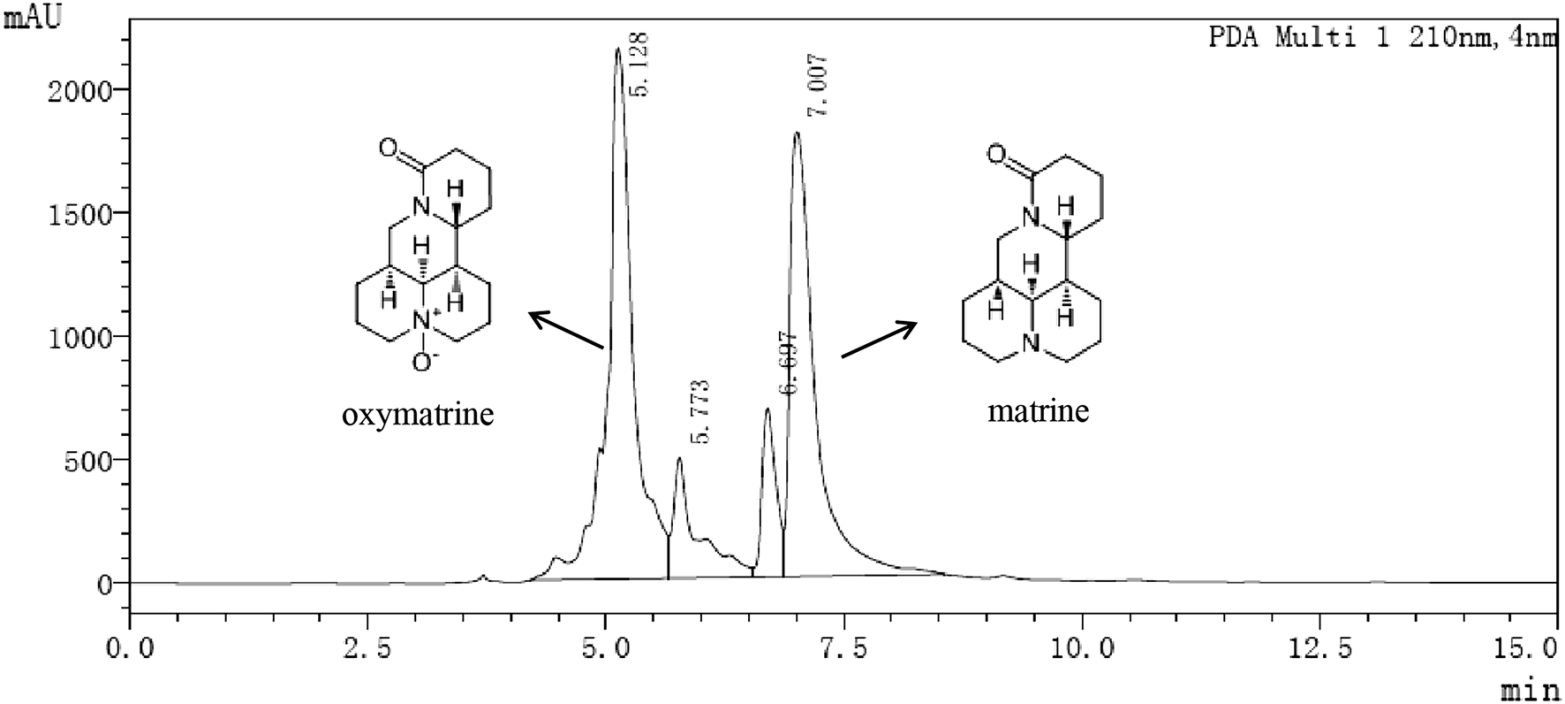
Chromatograms for quantifying the oxymatrine and matrine in *Sophora flavescens* Alkaloids (SFAs) at 210 nm. Peaks 1 was identified as oxymatrine (retentiontime, 5.128 min) and peaks 2 was matrine (retentiontime, 7.007 min)

Previous research results indicated that matrine and oxymatrine were the main active components in the SFAs. The content ratio of oxymatrine and matrine were analysed by matching retention times and the HPLC chromatograms is shown in Fig. 1. According to previous reports and corresponding standard compound, peaks 1 was identified as oxymatrine (retentiontime, 5.128 min) and peaks 2 was matrine (retentiontime, 7.007 min). Based on the standard curve of oxymatrine reference, the value of *F*_(*oxymatrine*)_ is 7,010,090, thus *y* = *F*_(*oxymatrine*)_ *x* + 5,621,990 (y is the peak area, and x is the concentration).In the same way, we calculated *F*_(*matrine*)_ is 8,198,700 and *y* = *F*_(*matrine*)_ *x* + 10,199,700. The final concentrations of oxymatrine is 4.840 ± 0.3005 mg/mL and matrine is 2.673 ± 0.2155 mg/mL, in other words, the ratio of oxymatrine and matrine in SFAs is 48.40 ± 3.05% and 26.73 ± 2.155% (Supplementary Table 1).

### Antimicrobial Susceptibility of Metronidazole Against 30 Clinical *Gardnerella vaginalis*

**Fig. 2:**
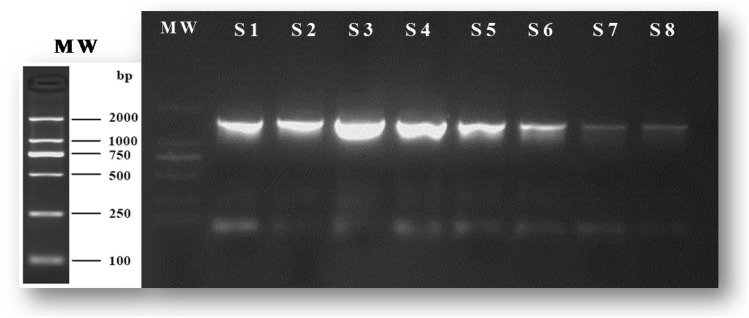
Representative electropherograms of 16S rDNA PCR products of partical*G. vaginalis. *MW: molecular weight standard; S1-S8: 8 strains of *G. vaginalis.* Expected amplicon size: 1400 bp

During the study period, a total of 30 isolates were isolated and detected, and the identity of presumptively isolated *G. vaginalis* was confirmed by PCR and 16S rDNA gene sequencing. 8 samples were taken for gel electrophoresis test to verify the accuracy of this PCR operation (Fig. 2), and all of the PCR amplification products were sent to Beijing SinoGenoMax Co., Ltd. for sequencing. Referencing to sequence alignment in GenBank and National Center for Biotechnology Information (NCBI), all of the 30 strains were *Gardnerella vaginalis* strain. According to the 2012 and 2018 Clinical and Laboratory Standards Institute (CLSI) guidelines for anaerobic drug sensitivity testing, MIC was determined using the broth microdilution method, with the standard strains of *Bacteroides fragilis* (ATCC 25285) as the quality control and *G. vaginalis* (ATCC 14018) served as the standard strains control. Please refer to the Supplementary Table 2 for MIC values of every clinical strain. Antimicrobial susceptibility testing results indicated that only 30% (9/30) clinical strains were sensitive to metronidazole, whereas the resistance rate was as high as 63.33% (19/30) (Table 1).

**Table 1:** Summary of the minimum inhibitory concentration and minimal bactericidal concentration concentrations of *Sophora flavescens* Alkaloids (SFAs) against 30 *G. vaginalis* strains of different susceptibility to metronidazole

Group	*N*, %	Metronidazole MIC Range(μg/mL)	SFAs MIC(mg/mL)		SFAs MBC(mg/mL)	
			Range	Mean ± SD	Range	Mean ± SD
metronidazole-sensitive	9, 30.00%	0.125–8.00	0.15625–0.625	0.2951 ± 0.0521	0.3125–1.250	0.5903 ± 0.219
metronidazole-intermediary	2, 6.67%	16	0.625	–	0.625–1.25	–
metronidazole-resistant	19, 63.33%	≥ 32	0.3125–2.5	0.9211 ± 0.546***	0.625–5	1.842 ± 1.092***

The *p*-value is calculated between the metronidazole-sensitive group and the metronidazole-resistant group.

***P < 0.001

**Table 2:** The inhibitory concentrations of metronidazole and SFAs against 4 metronidazole-resistant *G. vaginalis* strains

Strains	Metronidazole (μg /mL)			SFAs (mg/mL)		
	MIC	MBIC	MBIC/MIC	MIC	MBIC	MBIC/MIC
*G. vaginalis*7	> 128	512	< 4	0.3125	0.625	2
*G. vaginalis*9	> 128	512	< 4	0.3125	0.625	2
*G. vaginalis*12	> 128	256	< 2	1.25	1.25	1
*G. vaginalis*18	> 128	512	< 4	0.3125	0.625	2
*G. vaginalis* (ATCC 14018)	2	128	64	0.625	1.25	2

### The Antimicrobial Effects of SFAs on 30 *G. vaginalis* Clinical Strains

The Minimum Inhibitory Concentration (MIC) and Minimum Bactericidal Concentration (MBC) values of SFAs were detected as previous described. The results showed that the MIC range of total matrine against *G. vaginalis* strains was 0.1563–2.5 mg/mL, which was only 1/128-1/8 of the clinical dose (about 20 mg/mL).The MBC value was in the range of 0.3125–5.0 mg/mL, which was also far lower than the clinical dose, indicating that the SFAs had significant inhibitory and killing effects on clinical strains of *G. vaginalis* (Table 1). Contrast with metronidazole-sensitive strains, the resistant group showed prominently increased MIC value (0.259 ± 0.0521 *vs* 0.9211 ± 0.546, *P* < 0.001) and the MBC value (0.5903 ± 0.219 *vs* 1.8420 ± 1.0920, *P* < 0.001), which suggested that the metronidazole-resistant group may have some drug resistance characteristics.

### The Antimicrobial Activity of SFAs Against Metronidazole-Resistant *G. vaginalis* with Strong Biofilm Formation Ability

Many studies have reported that biofilm formation may increase drug resistance, thus 4 metronidazole-resistant strains which had strong biofilm formation ability were chosen to the next study. *G. vaginalis* planktonic and biofilm-associated growth was inhibited only when high concentrations of metronidazole were used (MIC ≥ 128 μg/mL, MBIC 256–512 μg/mL). Furthermore, the MIC of standard strains of *G. vaginalis* was 2 μg/mL and its MBIC increased to 128 μg/mL. And the MIC of SFAs was 0.3125–1.25 mg/mL, which is only 1/32-1/16 of the clinical dosage of the vaginal administration. The MBIC of the SFAs against *G. vaginalis* biofilm formation was 0.625–1.25 mg/mL, which was increased only one to two times as compared with the MIC. Moreover, the MBIC of SFAs against standard strains of *G. vaginalis* was 1.25 mg/mL, which was increased two times with MIC (0.0625 mg/mL) (Table 2). These results showed that, although the biofilm has formed, SFAs still exhibited a bactericidal effect to inhibit the continued growth and biofilm formation.

### Effect of *Sophora flavescens* Alkaloids on the Ultrastructural Morphology of *G. vaginalis* Biofilm by SEM

**Fig. 3:**
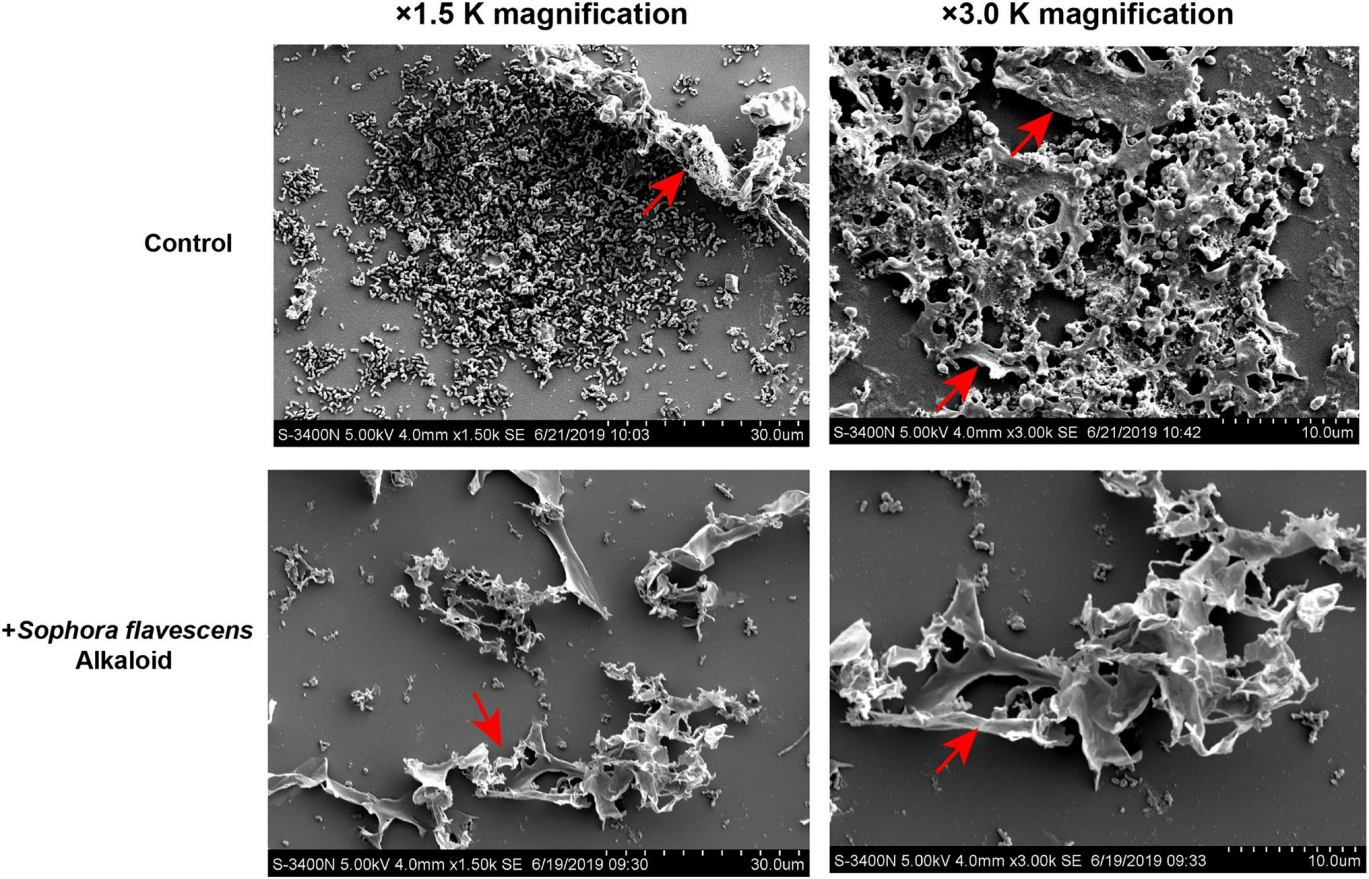
Effect of *Sophora flavescens* Alkaloids (SFAs) on the ultrastructural morphology of *G. vaginalis* biofilm by scanning electron microscopy. Morphology and ultrastructure of the *G. vaginalis* biofilm in the control group and 0.3125 mg/mL *Sophora flavescens* Alkaloids added group at 1.5 K and 3.0 K magnification. Red arrows indicate biofilm (Color figure online)

Furthermore, the effect of SFAs on *G. vaginalis* biofilm morphology was observed using a scanning electron microscope to illustrate the bactericidal mechanisam. The gradual formation of *G. vaginalis* biofilm was observed with increased time in culture. In the control group, *G. vaginalis* strains were primarily short bacillus with normal morphology at low magnification. As the incubation time increases, *G. vaginalis* single colonies were gathered in groups, and biofilm was preliminary form. These colonies grew continuously and started to fuse, followed by an increase in the secretion of intercellular matrixes. The thick biofilm showed dense bacterial clumps with a complex and stereo specific film structure, embedded in extracellular polymeric substances. However, in the *Sophora flavescens* Alkaloids added group, the number of bacteria was significantly reduced after the addition of SFAs and bacterial count declined sharply and altered *G. vaginalis* morphology was cataclastic. Due to the reduced secretion of intercellular matrixes, there was less biofilm formation, and the biofilm became discontinuous and fragmented (Fig. 3).These findings suggested that SFAs inhibited biofilm formation by destroying the *G. vaginalis* biofilm structure, thereby suppressing the growth of *G. vaginalis*.

## Discussion

BV is characterized by a relative decrease in beneficial lactobacillus and the significant increase in number of anaerobic bacteria *G. vaginalis* is frequently present in women and the detection rate of *G. vaginalis* was 87.5% in BV patients, 26.4% in healthy women, and 34.0% in women in an intermediate state [16]. In this study, a total of 76 clinical vaginal strains were isolated from 100 BV patients, and 30 strains were *G. vaginalis* after sequencing identification. As the predominant bacterial species, *G. vaginalis* can facilitate the growth of BV-related anaerobic bacteria by reducing H_2_O_2_, which can utilize metabolites of anaerobic bacteria and further raise the vaginal pH and reducing lactobacillus *G. vaginalis* inimitably has three toxic characteristics: cytotoxicity, vaginal epithelial adhesion, and biofilm formation, thus possesses higher virulence than other BV-associated bacteria[17, 18].

Metronidazole, a first-generation nitroimidazole, is effective against anaerobes by inhibiting nucleic acid synthesis and became the current treatment of choice for BV. However, with the widespread use of metronidazole in BV clinical patients, the rate of BV recurrence within three months was highly reached 58% with drug-resistant strains emerged [19]. In our study, we found 63.33% (19/30) of *G. vaginalis* clinical strains of BV patients exhibited resistance to metronidazole. The actual mechanism of resistance of metronidazole to *G. vaginalis* has not yet been fully elucidated, but the primary basis for resistance is decreased uptake of the drug or altered reduction efficiency [20]. The biofilm prevention and decreased activity of the nitroreductase lead to reduced uptake of the drug, and the other mechanisms include active efflux, inactivation of the drug, and increased DNA damage repair [21–23]. Therefore, controlling resistance to metronidazole and finding more effective drugs for BV requires prompt solutions.

*Sophora flavescens* is a traditional Chinese medicine, derived from the dried root of the legume plant *Sophora flavescens* Ait., which has been listed in the Pharmacopoia of the Peoples Republic of China for the treatment of dysentery, hematochezia, jaundice, oliguria, vulvarswelling, eczema, ulcers, scabies, and leprosy[9]. *Sophora flavescen*s Alkaloids (SFAs) is one of the active ingredients of *Sophora flavescen*s and some studies demonstrated that SFAs has a variety of antibacterial and anti-inflammatory activities and pharmacological antipruritic effects [9, 24, 25]. Matrine and oxymatrine was the active ingredient of SFAs, which could attenuation of acute lung injury and inhibit the progression of cancer cell lines in vitro [26, 27].Studies confirmed that matrine and oxymatrine inhibited growth of tumour in vivo of mice by regulating GADD45B, Bcl-2, and caspase-3, and also regulated anti-inflammatory responses through AGE expression and Nrf translocation and assist therapy for cardiovascular disease by affecting the JAK2/STAT3 and ATF6 signaling pathway [28-32]. Previous clinical studies demonstrated that SFAs can effectively treat BV and significantly improve clinical symptoms but the actual mechanism is unclear [33]. In this in vitro study, SFAs exhibited inhibitory and bactericidal effects on clinical metronidazole-resistant strains, and the MBC and MIC were much lower than the doses used clinically. This further substantiated use of SFAs as an option for clinical use.

It has been hypothesized that the biofilm establishment plays a key role in the pathogenesis of BV and increases the resistance to the host immune defense system and phagocytosis [34]. Swidsinsk et al. found that at 10–12 weeks after treatment of BV, *G. vaginalis*-dominated bacterial biofilm were detected in 40% of BV patients and concluded that the primary reason was the reactivation of biochemically inactivated biofilm, not the occurrence of a new infection [35]. *G. vaginalis* biofilm cannot be effectively cleared by the human immune system or completely inactivated by antibacterial drugs, therefore, remains chronic and persistent infections [36]. Studies have shown that the antimicrobial response is significantly different which is associated with a planktonic or biofilm-associated style. The slow or no growth allows bacteria in biofilm to be safe from antibacterial drugs, thereby reducing their sensitivity to these drugs. Also, the biofilm matrix can serve as a barrier that reduces the penetration of antibacterial drugs [37].Therefore, *G. vaginalis* in a biofilm can tolerate high concentration of H_2_O_2_ and lactic acid against planktonic style [38].In this study, it was found that the inhibitory concentration of metronidazole against standard strains of *G. vaginalis* was increased by 63 times due to biofilm formation. The inhibitory concentration of SFAs against biofilm was increased by only onefold compared with MIC, which suggested that SFAs could effectively inhibit *G. vaginalis*in biofilm-associated form. Furthermore, the inhibition of biofilm and ultrastructure changes of *G. vaginalis* biofilm morphology was also observed by transmission electron microscope.

Even taking in consideration the limited survey samples the study, current data suggest SFAs could not only inhibit the growth of metronidazole-resistant *G. vaginalis* in planktonic and biofilm levels, but also destroyed the biofilm formation and microstructure. According to previous results, the antibiotic tolerance of *G. vaginalis* and recurrence of BV is associated with the ability of biofilm formation, thereby, identifying more novel therapeutics that target vaginal biofilm may contributed to the prevention of BV recurrence[39].

## Conclusions

In this study, we identified 30 clinical *G. vaginalis* strains from the vaginal secretions of BV patients, and 19 of which were resistant to metronidazole. We also evaluated effect of *Sophora flavescens* Alkaloids on *G. vaginalis*, and found that *Sophora flavescens* Alkaloids could inhibit the growth of *G.vaginal*even for drug-resistant strains. *Sophora flavescens* Alkaloids also inhibited the *G. vaginalis* biofilm formation by destroying the microstructure, thus thinning the thick biofilm. In summary, *Sophora flavescens* Alkaloids could not only inhibit the growth of metronidazole-resistant *G. vaginalis* in planktonic and biofilm levels, but also destroyed the biofilm morphology and microstructure, which may contribute to the prevention of BV recurrence.

## Supplementary Information

Below is the link to the electronic supplementary material.

Supplementary file1 (DOCX 33 KB)

## Data Availability

All data have been submitted in the paper.

## References

[1] Janulaitiene M, Paliulyte V, Grinceviciene S (2017). Prevalence and distribution of Gardnerella vaginalis subgroups in women with and without bacterial vaginosis. BMC Infect Dis.

[2] Nelson DB, Macones G (2002). Bacterial vaginosis in pregnancy:current findings and future directions. Epidemiol Rev.

[3] Sherrard J, Wilson J, Donders G (2018). 2018 European (IUSTI/WHO) International union against sexually transmitted infections (IUSTI) World Health Organisation (WHO) guideline on the management of vaginal discharge. Int J STD AIDS.

[4] Schwebke JR, Muzny CA, Josey WE (2014). Role of Gardnerella vaginalis in the pathogenesis of bacterial vaginosis: a conceptual model. J Infect Dis.

[5] Onderdonk AB, Delaney ML, Fichorova RN (2016). The human microbiome during bacterial vaginosis. Clin Microbiol Rev.

[6] Patterson JL, Stull-Lane A, Girerd PH (2010). Analysis of adherence, biofilm formation and cytotoxicity suggests a greater virulence potential of Gardnerella vaginalis relative to other bacterial vaginosis-associated anaerobes. Microbiology.

[7] Turovskiy Y, Sutyak Noll K, Chikindas ML (2011). The aetiology of bacterial vaginosis. J Appl Microbiol.

[8] Alves P, Castro J, Tatiana CS, Cereija B (2014). Gardnerella Vaginalis outcompetes 29 other bacterial species isolated from patients with bacterial vaginosis, using in an in vitro biofilm formation model. J Infect Dis.

[9] He X, Fang J, Huang L (2015). Sophora flavescens Ait.: traditional usage, phytochemistry and pharmacology of an important traditional Chinese medicine. J Ethnopharmacol.

[10] Xiu W, Jianchun L, Yuzhen H (2017). Effect of Sophora flavescens alkaloid on aerobic vaginitis in gel form for local treatment. J Tradit Chin Med.

[11] Jia F, Zhou Q, Li X (2019). Total alkaloids of Sophora alopecuroides and matrine inhibit auto-inducer 2 in the biofilm of Staphylococcus epidermidis. Microb Pathog.

[12] Clinical and Laboratory Standards Institute (2012) Methods for Antimicrobial Susceptibility Testing of Anaerobic Bacteria; Approved Standard, 8, Clinical and Laboratory Standards Institute, Wayne, PA, CLSI Document M11-A-8.

[13] Clinical and Laboratory Standards Institute (2018) Standards for Antimicrobial Susceptibility Testing. 28 Clinical and Laboratory Standards Institute, Wayne, PA, CLSI Supplement M100eS28.

[14] Stepanovic S, Vukovic D, Dakic I (2000). A modified microtiter-plate test for quantification of staphylococcal biofilm formation. J Microbiol Methods.

[15] Zayed SM, Aboulwafa MM, Hashem AM, Saleh SE (2021). Biofilm formation by Streptococcus mutans and its inhibition by green tea extracts. AMB Express.

[16] Noll KS, Prichard MN, Khaykin A (2012). The natural antimicrobial peptide subtilosin acts synergistically with glycerol monolaurate, lauric arginate, and ε-poly-L-lysine against bacterial vaginosis-associated pathogens but not human lactobacilli. AAntimicrob Agents Chemother.

[17] Mendling W (2016). Vaginal Microbiota. Adv Exp Med Biol.

[18] Patterson JL, Stull-Lane A, Girerd PH (2010). Analysis of adherence, biofilm formation and cytotoxicity suggests a greater virulence potential of Gardnerella vaginalis relative to other bacterial-vaginosis-associated anaerobes. Microbiology.

[19] Aroutcheva AA, Simoes JA, Behbakht K (2001). Gardnerella vaginalis isolated from patients with bacterial vaginosis and from patients with healthy vaginal ecosystems. Clin Infect Dis.

[20] Löfmark S, Edlund C, Nord CE (2010). Metronidazole is still the drug of choice for treatment of anaerobic infections. Clin Infect Dis.

[21] Algburi A, Zhang Y, Weeks R (2017). Gemini cationic amphiphiles control biofilm formation by bacterial vaginosis pathogens. Antimicrob Agents Chemother.

[22] Land KM, Johnson PJ (1999). Molecular basis of metronidazole resistance in pathogenic bacteria and protozoa. Drug Resist Updat.

[23] Löfmark S, Fang H, Hedberg M, Edlund C (2005). Inducible metronidazole resistance and nim genes in clinical Bacteroides fragilis group isolates. Antimicrob Agents Chemother.

[24] Lee JH, Shin H, Kim YJ (2014). Pseudomonas aeruginosa-induced IL-1beta production is inhibited by Sophora flavescens via the NF-kappaB/inflammasome pathways. J Microbiol.

[25] Cha SM, Cha JD, Jang EJ (2016). Sophora flavanone G prevents Streptococcus mutans surface antigen I/II-induced production of NO and PGE2 by inhibiting MAPK-mediated pathways in RAW 264.7 macrophages. Arch Oral Biol.

[26] Xu GL, Yao L, Rao SY (2005). Attenuation of acute lung injury in mice by oxymatrine is associated with inhibition of phosphorylated p38 mitogen-activated protein kinase. J Ethnopharmacol.

[27] Jiang H, Meng F, Li J (2005). Anti-apoptosis effects of oxymatrine protect the liver from warm ischemia reperfusion injury in rats. World J Surg.

[28] Huang H, Wang Q, Du T (2018). Matrine inhibits the progression of prostate cancer by promoting expression of GADD45B. Prostate.

[29] Gu YY, Chen MH, May BH (2018). Matrine induces apoptosis in multiple colorectal cancer cell lines in vitro and inhibits tumour growth with minimum side effects in vivo via Bcl-2 and caspase-3. Phytomedicine.

[30] Zhang Y, Yang X, Qiu C (2018). Matrine suppresses AGE-induced HAEC injury by inhibiting ROS-mediated NRLP3 inflammasome activation. Eur J Pharmacol.

[31] Liu Z, Zhang Y, Tang Z (2017). Matrine attenuates cardiac fibrosis by affecting ATF6 signaling pathway in diabetic cardiomyopathy. Eur J Pharmacol.

[32] Zhao XB, Qin Y, Niu YL (2018). Matrine inhibits hypoxia/reoxygenation-induced apoptosis of cardiac microvascular endothelial cells in rats via the JAK2/STAT3 signaling pathway. Biomed Pharmacother.

[33] Huilan Du, Shaobin W, Yong T (2020). Guidance on clinical application of Sophora flavescens gel. Chin Tradit Herbal Drugs.

[34] Hardy L, Cerca N, Jespers V (2017). Bacterial biofilm in the vagina. Res Microbiol.

[35] Verstraelen H, Swidsinski A (2013). The biofilm in bacterial vaginosis: implications for epidemiology, diagnosis and treatment. Curr Opin Infect Dis.

[36] Patterson JL, Girerd PH, Karjane NW (2007). Effect of biofilm phenotype on resistance of Gardnerella vaginalis to hydrogen peroxide and lactic acid. Am J Obstet Gynecol.

[37] Swidsinski A, Dörffel Y, Loening-Baucke V (2011). Response of Gardnerella vaginalis biofilm to 5 days of moxifloxacin treatment. FEMS Immunol Med Microbiol.

[38] Roilides E, Simitsopoulou M, Katragkou A (2015). How biofilms evade host defenses. Microbiol Spectr.

[39] Linyuan Fan, Zhaohui liu, Zhan Zhang, et al (2022) Research Square [Preprint].Doi:10.21203/rs.3.rs-354686/v1. Available from: https://www.researchsquare.com/article/rs-354686/v1

