# Supplementary material for: Antimicrobial Effects of Sophora flavescens Alkaloids on Metronidazole-Resistant Gardnerella vaginalis in Planktonic and Biofilm Conditions
Source: Curr Microbiol. 2023 Jun 29;80(8):263. doi: 10.1007/s00284-023-03378-x (PMC10310575; doi:10.1007/s00284-023-03378-x)
Supplement: Supplementary file 1 — Supplementary file1 (DOCX 33 KB) [file 284_2023_3378_MOESM1_ESM.docx]

**CURRENT MICROBIOLOGY**

**Antimicrobial effects of *Sophora flavescens Alkaloids* on metronidazole-resistant *Gardnerella vaginalis* in planktonic and biofilm conditions**

Linyuan Fan^1^, Zhaohui Liu^1*^, Zhan Zhang^1^, Huihui Bai^2^

1 Department of Gynecology, Beijing Obstetrics and Gynecology Hospital, Capital Medical University, Beijing Maternal and Child Health Care Hospital. Beijing 100026, China

2 Department of Clinical Laboratory, Beijing Obstetrics and Gynecology Hospital, Capital Medical University, Beijing Maternal and Child Health Care Hospital. Beijing 100026, China

**Correspondence**: Prof. Zhao-Hui Liu, Department of Gynecology, Beijing Obstetrics and Gynecology Hospital, Capital Medical University, Beijing Maternal and Child Health Care Hospital. NO.251, Yaojiayuan Road, Chaoyang District, Beijing, China, 100026 .Tel: +86-010-52272053

E-mail: [liuzhaohui@ccmu.edu.cn](mailto:liuzhaohui@ccmu.edu.cn)

**Supplementary Table 1.** The peak area of HPLC and final calculated concentration of oxymatrine and matrine

| **Sample** | **Oxymatrine Area** | **Oxymatrine Concentration**  **(mg/mL)** | **Matrine Area** | **Matrine Concentration (mg/mL)** |
| --- | --- | --- | --- | --- |
| **Sample 1** | 41713562 | 5.15 | 33473267 | 2.84 |
| **Sample 2** | 37512681 | 4.55 | 32770519 | 2.75 |
| **Sample 3** | 39426564 | 4.82 | 30135852 | 2.43 |

**Supplementary Table 2.** The minimum inhibitory concentration and minimal bactericidal concentration concentrations of 30 *G.vaginalis* strains to metronidazole and *Sophora flavescens* Alkaloids

| **Isolate No.** | **Metronidazole (μg/mL)** | | **SFAs(mg/mL)** | |
| --- | --- | --- | --- | --- |
|  | **MIC** | **MBC** | **MIC** | **MBC** |
| Clinical strain 1 | ＞128 | ＞128 | 1.25 | 2.5 |
| Clinical strain 2 | 8 | 16 | 0.3125 | 0.625 |
| Clinical strain 3 | 8 | 16 | 0.3125 | 0.625 |
| Clinicalstrain 4 | 16 | 32 | 0.625 | 1.25 |
| Clinical strain 5 | ＞128 | ＞128 | 0.625 | 1.25 |
| Clinical strain 6 | ＞128 | ＞128 | 1.25 | 2.5 |
| Clinical strain 7 | ＞128 | ＞128 | 0.3125 | 0.625 |
| Clinical strain 8 | 16 | 32 | 0.625 | 0.625 |
| Clinical strain 9 | ＞128 | ＞128 | 0.3125 | 0.625 |
| Clinical strain 10 | 8 | 16 | 0.3125 | 0.625 |
| Clinical strain 11 | 8 | 64 | 0.15625 | 0.3125 |
| Clinical strain 12 | ＞128 | ＞128 | 1.25 | 2.5 |
| Clinical strain 13 | <0.125 | 0.125 | 0.3125 | 0.625 |
| Clinical strain 14 | 8 | 64 | 0.3125 | 0.625 |
| Clinical strain 15 | ＞128 | ＞128 | 0.625 | 1.25 |
| Clinical strain 16 | ＞128 | ＞128 | 0.625 | 1.25 |
| Clinical strain 17 | ＞128 | ＞128 | 0.3125 | 0.625 |
| Clinical strain 18 | ＞128 | ＞128 | 0.3125 | 0.625 |
| Clinical strain 19 | ＞128 | ＞128 | 2.5 | 5 |
| Clinical strain 20 | ＞128 | ＞128 | 1.25 | 2.5 |
| Clinical strain 21 | ＞128 | ＞128 | 1.25 | 2.5 |
| Clinical strain 22 | ＞128 | ＞128 | 0.625 | 1.25 |
| Clinical strain 23 | 2 | 4 | 0.3125 | 0.625 |
| Clinical strain 24 | ＞128 | ＞128 | 1.25 | 2.5 |
| Clinical strain 25 | ＞128 | ＞128 | 0.625 | 1.25 |
| Clinical strain 26 | <0.125 | 0.125 | 0.3125 | 0.625 |
| Clinical strain 27 | ＞128 | ＞128 | 1.25 | 2.5 |
| Clinical strain 28 | 2 | 4 | 0.3125 | 0.625 |
| Clinical strain 29 | ＞128 | ＞128 | 1.25 | 2.5 |
| Clinical strain 30 | 32 | ＞128 | 0.625 | 1.25 |
| ATCC 14018 | 2 | 4 | 0.625 | 1.25 |
